# Sintering Bonding of SiC Particulate Reinforced Aluminum Metal Matrix Composites by Using Cu Nanoparticles and Liquid Ga in Air

**DOI:** 10.3390/nano11071800

**Published:** 2021-07-10

**Authors:** Zeng Gao, Congxin Yin, Dongfeng Cheng, Jianguang Feng, Peng He, Jitai Niu, Josip Brnic

**Affiliations:** 1 School of Materials Science and Engineering, Henan Polytechnic University, Jiaozuo 454003, China; gaozeng@hpu.edu.cn (Z.G.); Ycx7469@163.com (C.Y.); jayfeng_cool@126.com (J.F.); 2 State Key Laboratory of Advanced Welding and Joining, Harbin Institute of Technology, Harbin 150001, China; hepeng@hit.edu.cn (P.H.); niujitai@163.com (J.N.); 3 Faculty of Engineering, University of Rijeka, 51000 Rijeka, Croatia; brnic@riteh.hr

**Keywords:** sintering bonding, Cu nanoparticles, liquid Ga, aluminum metal matrix composites, gas tightness

## Abstract

SiC particulate reinforced aluminum metal matrix composites (SiC_p_/Al MMCs) are characterized by controllable thermal expansion, high thermal conductivity and lightness. These properties, in fact, define the new promotional material in areas and industries such as the aerospace, automotive and electrocommunication industries. However, the poor weldability of this material becomes its key problem for large-scale applications. Sintering bonding technology was developed to join SiC_p_/Al MMCs. Cu nanoparticles and liquid Ga were employed as self-fluxing filler metal in air under joining temperatures ranging from 400 °C to 500 °C, with soaking time of 2 h and pressure of 3 MPa. The mechanical properties, microstructure and gas tightness of the joint were investigated. The microstructure analysis demonstrated that the joint was achieved by metallurgical bonding at contact interface, and the sintered layer was composed of polycrystals. The distribution of Ga was quite homogenous in both of sintered layer and joint area. The maximum level of joint shear strength of 56.2 MPa has been obtained at bonding temperature of 450 °C. The specimens sintering bonded in temperature range of 440 °C to 460 °C had qualified gas tightness during the service, which can remain 10^−10^ Pa·m^3^/s.

## 1. Introduction

Certain properties of SiC particulate reinforced aluminum metal matrix composites (SiC_p_/Al MMCs), like controllable thermal expansion, high thermal conductivity, etc., promote this material as very acceptable in various applications [1,2,3,4]. However, bonding of SiC_p_/Al MMCs is still one of the unsettled problems which limit widespread use of considered materials in structural and functional applications [5]. A benefit from vacuum stirring casting technology, 15 vol.% SiC_p_/Al MMCs can be mass produced with lower production costs compared to the other production technology, such as powder metallurgic method and liquid impregnation technology. Following the previous bonding technology of aluminum alloy, most researches have been conducted on fusion welding, using arc, laser and electron beam as heating sources to join SiC_p_/Al MMCs [6,7,8,9]. However, attempts to weld this type create some practical problems [10]. The first limitation is the chemical reactions in the matrix/reinforcement interfaces initiated by high temperature. In this particular case, this SiC reinforcement will react with the aluminum in the welding following Equation (1):

3SiC + 4Al = 3Si + Al_4_C_3_, (1)

The chemical product Al_4_C_3_ degrades the mechanical properties of joints due to its brittleness and inhomogeneous distribution [11]. Another limitation is the distribution of reinforcement particles. During fusion welding, there is a tendency to disturb the distribution of SiC particles in the molten pool and may disappear completely, even MMCs are used as filler metal [12]. In addition to this, the poor fluidity of the molten pool may cause the welding defects, such as pore, slag inclusions and incomplete backfill, etc. [6,13]. In addition to fusion welding, some other welding techniques can be applied to SiC_p_/Al MMCs bonding, such as solid-state diffusion bonding, brazing, friction stir welding, etc. However, these welding methods involve several difficulties to join SiC_p_/Al MMCs in practical applications. For example, solid-state diffusion bonding of such composites results in excessive plastic deformation under high applied pressure [14]. Given the brazing technique, it is difficult to develop the filler metal that can very well wet both the aluminum matrix and SiC particles due to the difference in physical and chemical properties of base metal and ceramic reinforcement [15]. Friction stir welding of SiC_p_/Al MMCs causes serious tool wear due to the existence of wear-resistant SiC particles in aluminum. In addition, it is easy to produce tunnel defects inside of the weld during the friction stir welding process [16].

In the present study, a preliminary research was conducted to investigate the sintering bonding performance of SiC_p_/Al MMCs using Cu nanoparticles (Cu NPs) and liquid Ga in an atmospheric environment. Ga can eliminate the aluminum oxide and form a low-temperature eutectic liquid phase with aluminum at temperature of 26.6 °C [17,18]. Meanwhile, the peritectic reaction between Ga and Cu occurs at temperature of 254 °C, as known from Cu-Ga binary phase diagram. Cu nanoparticles are of great interest due to their potential applications in the fields of electronic packaging [19]. Reducing the size of Cu particles to nanoscale, their characteristics can vary greatly from those of bulk state. Typically, the decrease of melting point and increase of diffusion coefficient are remarkable compared with that of bulk state [20,21,22,23]. Based on the character of liquid Ga and Cu nanoparticles, it would be promising to use liquid Ga and Cu nanoparticle as filler metal to join SiC_p_/Al MMCs without flux [24]. Furthermore, the understanding of sintering bonding, interaction between liquid Ga and Cu nanoparticles is still limited. In this work, different temperatures were considered to evaluate the influence of nanoparticles on joint microstructure, mechanical properties and gas tightness.

## 2. Materials and Methods

**Figure 1:**
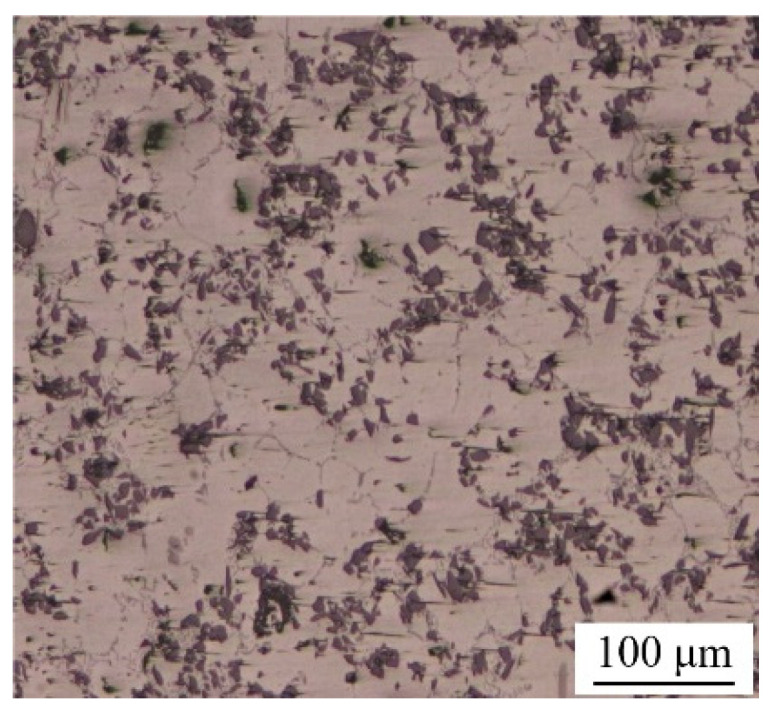
Metallographic structure of 15 vol.% SiC_p_/6063 Al MMCs manufactured by vacuum stirring casting method.

In this research, the material to be bonded was 6063 aluminum matrix composites reinforced with 15 vol.% SiC particles (15 vol.% SiC_p_/6063 Al MMCs), which was commercially manufactured by vacuum stirring casting method. SiC_p_/6063 Al MMCs is widely used in aerospace industry, electronic packaging, automotive industry, etc. The density of fabricated 15 vol.% SiC_p_/6063 Al MMCs was measured to be 2.82 g/cm^3^. The microstructure of as-received 15 vol.% SiC_p_/6063 Al MMCs mainly consists of α-Al and SiC particles with sizes between 10 and 25 μm, as shown in Figure 1. Due to the characteristics of solidification dynamics of liquid metal, SiC particles are mainly distributed on α-Al grain boundaries during the manufacturing process. The properties of this material will be improved because of the effect of fine grain strengthening and dislocation strengthening. The solid-liquidus temperature of 15 vol.% SiC_p_/6063 Al MMCs is in the range of 580–639 °C. Cu nanoparticles (Cu NPs) were purchased from the commercial company. The size of Cu NPs is around 50 nm. Ga is a low melting point metal, with a melting point of 29.7 °C, which is much lower than that of In (156.6 °C) and Sn (231.9 °C). In addition, Ga has excellent wetting properties on glass and ceramics. In this research, the purity of Ga and Cu NPs was 99.99% and 99.9%, respectively. All chemical reagents used in this experiment were of analytical grade and without any subsequent treatment.

**Figure 2:**
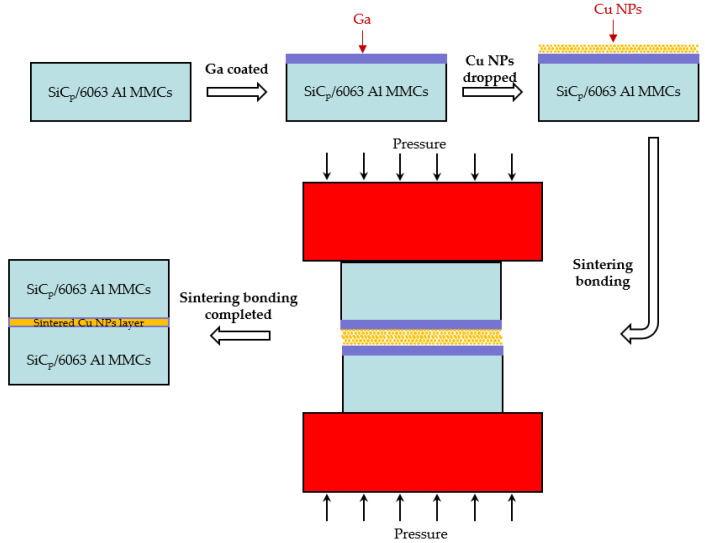
Schematic representation of sintering bonded SiC_p_/6063 Al MMCs joint.

**Figure 3:**
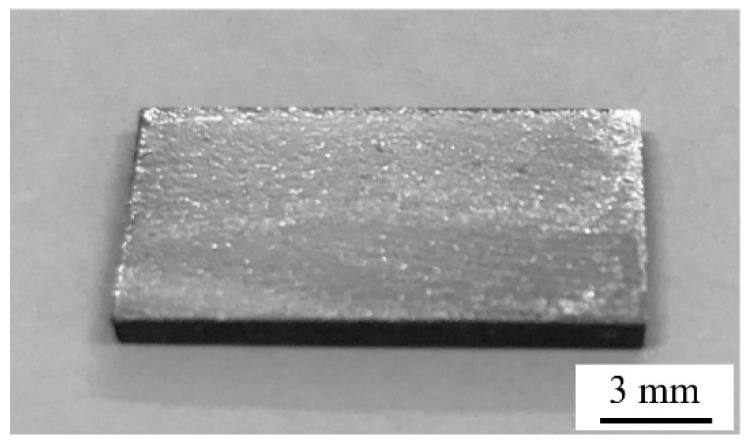
Photograph of 15 vol.% SiC_p_/6063 Al MMCs after Ga coating on bonding surface.

For the purpose of sintering bonding experiment, samples of SiC_p_/6063 Al MMCs were cut into pieces of 10 × 15 × 2 mm^3^. The bonding surface of the specimen was mechanically polished on 1000 grit grinding paper and then cleaned by ultrasonic cleaners with ethanol for 5 min. The schematic representation of sintering bonded SiC_p_/6063 Al MMCs joint is shown in Figure 2. In order to break the oxidation film on SiC_p_/6063 Al MMCs and improve the bonding properties, a liquid 10 μm thick Ga (equal to 5.9 mg/cm^2^) was lightly smeared on the bonding surface by using a warmed (about 40–60 °C) soft cloth impregnated with liquid Ga. The thickness of Ga was controlled by high precision electronic scale. Figure 3 is a photograph of 15 vol.% SiC_p_/6063 Al MMCs after coating Ga on the bonding surface. As can be seen, Ga is uniformly coated over the surface of the composites. Thereafter, the Ga-coated specimen was quickly transferred to a dry and low temperature environment to solidify the Ga liquid, minimizing intergranular permeability and the solution between Ga and SiC_p_/6063 Al MMCs. Cu NPs were uniformly dropped on solidified Ga layer. The mass ratio of Ga and Cu was controlled to be 2:1. The binary phase diagram of Cu-Ga demonstrated that these two elements can interact with each other to produce solid solution and intermetallic compounds such as Cu_3_Ga, Cu_2_Ga, Cu_5_Ga_3_, Cu_3_Ga_2_ and CuGa_2_, depending on the temperature and concentration. The two specimens after Cu NPs dropping were assembled together and then pressed by a preheated steel plate. The sintering bonding process was performed in a resistance furnace for 2 h under a pressure of 3 MPa. During sintering bonding process, rapid heating of the sample is the basic requirement of this experiment since any delay of heating may cause the intergranular diffusion of Ga into SiC_p_/6063 Al MMCs though grain and phase boundary. The rapid heating process will result in a solid solution of Ga into Al and Cu to avoid intergranular permeability.

Before sintering bonding, the morphological features of Cu NPs were observed by scanning electron microscope (SEM, Carl Zeiss NTS GmbH, Merlin Compact, Jena, Germany). Thermodynamic characteristics of Cu NPs were measured by differential scanning calorimetry (DSC, Q100, TA Instruments, New Castle, DE, USA) at a heating rate of 10 °C/min. After sintering bonding, the shear strength of the lapped joint was evaluated by an electronic universal testing machine (CMT5205, MTS Systems (China) Co. Ltd., Shenzhen, China) with the constant shear rate of 0.2 mm/min. At least 5 sintering bonded specimens related to each group were used for the test, and the average test value was calculated. In addition to that, Vickers hardness (HBRV-187.5, Light-Mach Tech. Co., Ltd., Shanghai, China) was tested near the joint. The morphological character of the sintering bonded interface was investigated by scanning electron microscope (SEM) and optical microscope (OLYMPUS GX51, Olympus Corporation, Tokyo, Japan). X-ray diffraction (XRD, Dmax-RB, Rigaku Corporation, Tokyo, Japan) was utilized to analyze the phase composition in joint fracture. Helium leak mass spectrometer ZQJ-530 (KYKY Technology Development Ltd., Beijing, China) was employed to investigate the gas tightness of the joint.

## 3. Results

### 3.1. Characteristics of Cu Nanoparticles

**Figure 4:**
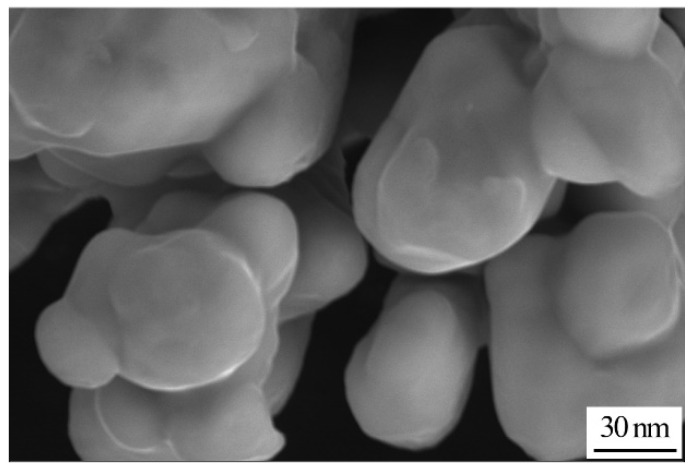
SEM image of Cu nanoparticles.

Figure 4 shows the SEM image of Cu NPs. As can be seen in Figure 4, the morphology of Cu NPs is presented in the form of quasi-spherical shapes. Moreover, Cu NPs are homogeneously dispersed without hard agglomerates. The average size of Cu NPs is around 50 nm.

**Figure 5:**
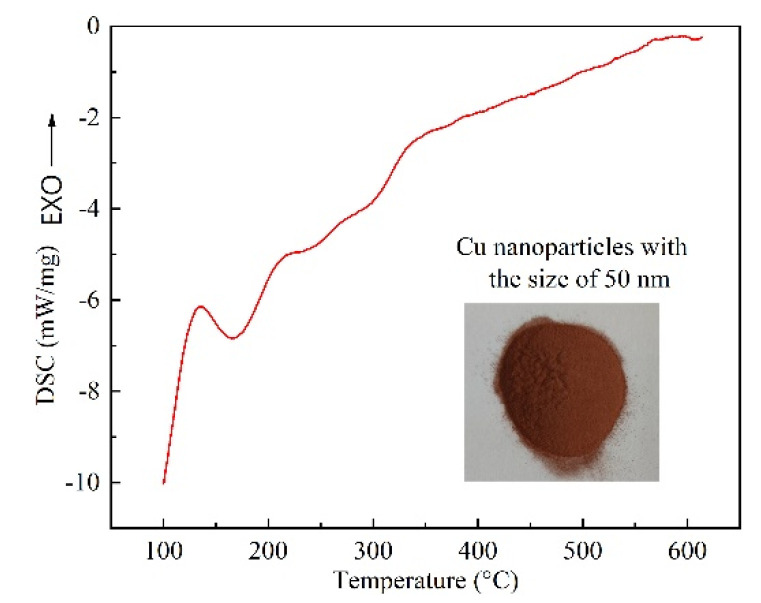
DSC curve of Cu nanoparticles.

As is known, the atomic arrangement on surface of nanoparticles varies greatly from the complete lattice inside the crystal, and the atoms at the interface have higher free energy. As a result, nanomaterials are metastable, which is quite sensitive to ambient temperature. To decrease the free energy of the system, atoms on the surface of nanoparticles will experience structural rearrangement and relaxation as the environmental temperature increases [25,26,27]. Figure 5 shows the DSC analysis of Cu NPs utilized in the experiment. For Cu NPs, a sharp exothermic peak between 120 °C and 160 °C is found in Figure 5. The second exothermic peak is quite broad compared with the first one, which is between 200 °C to 320 °C, implying the occurrence of a relatively uniform heat flow. The reason for this broad uniformity is that the Cu NPs have gone through a quite mild ripening process, which is a slow exothermic process. Different from the smooth DSC curves of bulk Cu before melting, there is exothermic peaks on DSC curve of Cu NPs prior to melting point of 1083.4 °C. These low-temperature exothermic peaks correspond to the enthalpy release of nanocrystals. The slower the heating rate is, the more heat is released.

In DSC test of metal particles, three types of phenomena can be responsible for the appearance of an exothermic peak, namely, the mutual diffusion of highly unstable atoms on the surface of metal particles during the sintering process, crystallization of amorphous metal particles and recrystallization of strained metal particles by heating. Therefore, in the case of sintering bonding in this research, the appearance of sintering necks by using Cu NPs is the main reason for the exothermic peak. As a result, the DSC analysis suggests that bonding temperature higher than 320 °C can assist the sintering joint performance. According to the thermodynamic melting process, Couchmann and Jesser proposed a relationship between the melting point and the solid particle size dimension, which is expressed as follows [25]:

(2) $$T_{m}=T_{m}^{e}[1-2\;\sigma_{\mathrm{sl}}\cos\theta \;{\left(L\rho_{s}r\right)}^{-1}],$$

where *T_m_* and $T_{m}^{e}$ are the melting point of nano material and bulk material, *σ_sl_* the interfacial energy between solid and liquid metal, *L* the latent heat of fusion, *ρ_s_* the density of solid metal, *r* the size of particle, *θ* the contact angle between embedded particle and matrix. Obviously, cos*θ* equal to 1 for the free particles. Equation (2) indicates that the melting point of metal will change as the particle size changes. The change of melting point (*T_m_*–$T_{m}^{e}$) is linearly related to the reciprocal of particle size. Therefore, the smaller the nanoparticle size is, the lower the sintering bonding temperature is. The sintering performance of Cu NPs is one of the key factors for achieving high strength bonding.

### 3.2. Microstructure Analysis of the Joint and XRD Analysis of the Fracture

**Figure 6:**
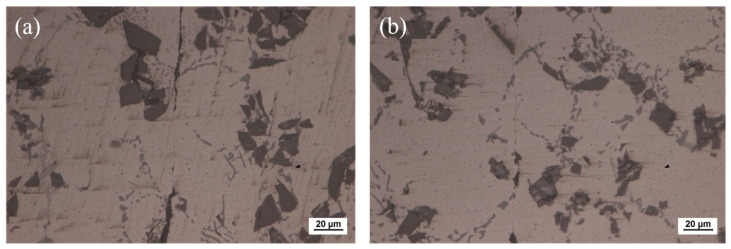

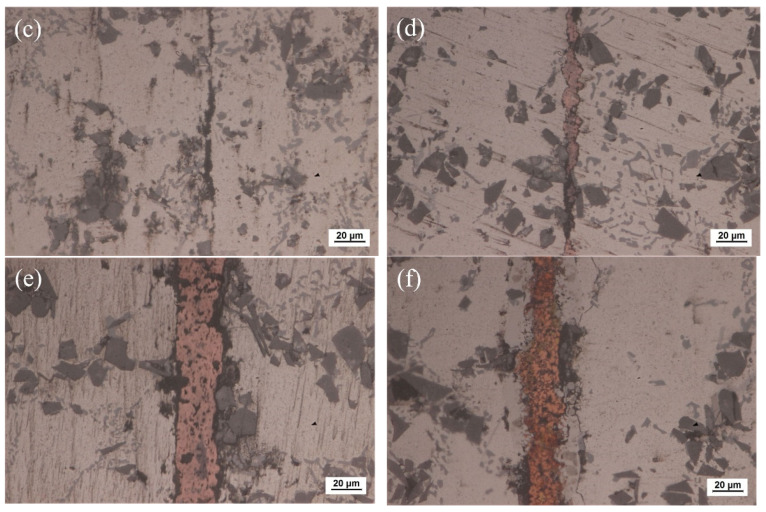
Optical microscope of the joints bonded at: (**a**) 440 °C using liquid Ga; (**b**) 450 °C using liquid Ga; (**c**) 460 °C using liquid Ga; (**d**) 440 °C using Cu NPs and liquid Ga; (**e**) 450 °C using Cu NPs and liquid Ga; (**f**) 460 °C using Cu NPs and liquid Ga.

The optical microscope image of SiC_p_/6063 Al MMCs joints after bonding is presented in Figure 6. Since liquid Ga was reported to be effective for bonding of aluminum [28,29], the bonding of SiC_p_/6063 Al MMCs was carried out by using liquid Ga at different temperatures. As shown in Figure 6a–c, the bonding of SiC_p_/6063 Al MMCs was realized by using only liquid Ga. Most areas are perfectly bonded with the assistance of liquid Ga. As is known from the Al-Ga binary phase diagram, Ga has the maximum solid solubility in aluminum at the temperature of 26.6 °C, which is around 20.0% (wt.%). The value of solid solubility decreases regardless of the increase or decrease in temperature. As a result, the width of the bonding area was quite small. However, some Ga cannot diffuse into aluminum, as shown in the black area along the bonded line due to the block of a small number of SiC particles and discontinuous oxide layer at the interface. In the area of bonding, these residual Ga are a type of defect, that is unfavorable to the strength of the joints. Figure 6d–f show the joint microstructure sintering bonded with Cu NPs and liquid Ga at temperatures that varied from 440 °C to 460 °C. As can be seen, the bonding of SiC_p_/6063 Al MMCs was successfully achieved by using Cu NPs and liquid Ga. Compared with the joint bonded by liquid Ga, the width of the bonding area is much wider due to the existence of the Cu layer. Figure 6d,e show that the morphology of Cu NPs disappears totally, leaving typical sintering characteristics such as micro-voids in the bonding area. Almost the whole nanoparticles fuse together to form continuous sintering Cu layer. However, the Cu NPs sintering bonded at 460 °C shows a different microstructure in the joint, showing isolated Cu particles of relatively large size, as shown in Figure 6f. In the bonding region, the size of Cu particles is much smaller than that of 450 °C, which leads to a fine, brittle structure. At higher sintering bonding temperature, the Cu NPs experience a higher degree of oxidation than that at lower temperature during the bonding, resulting in a sintering barrier for Cu NPs.

**Figure 7:**
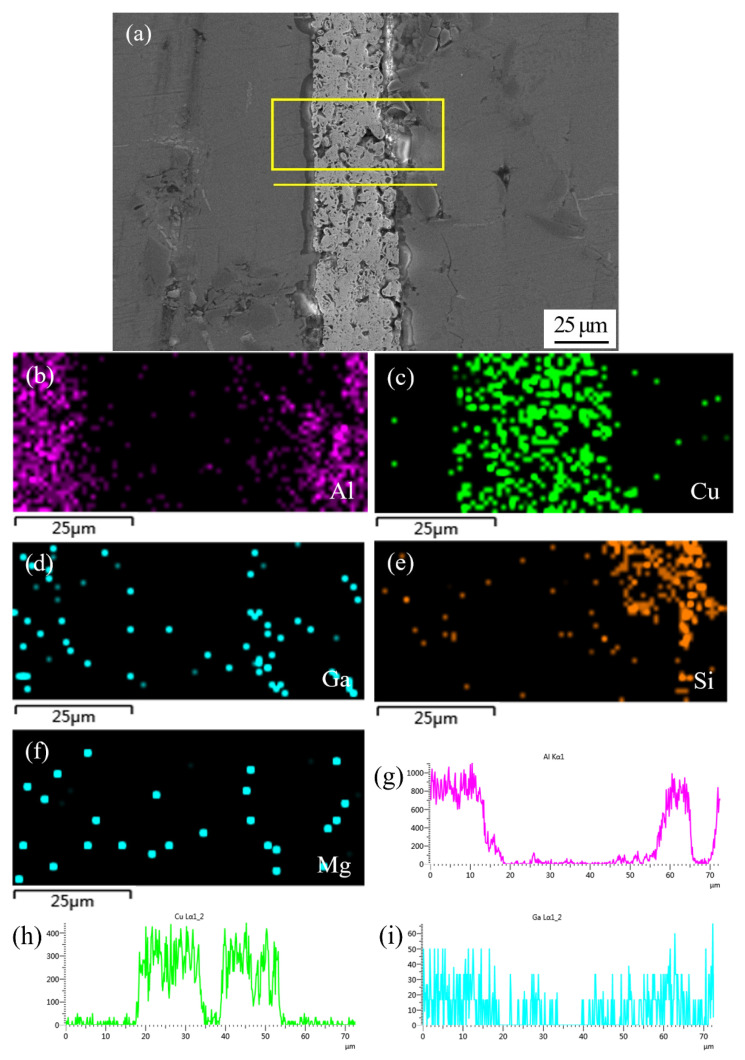
SEM micrograph at the interface of SiC_p_/6063 Al MMCs joint sintering bonded at 450 °C using Cu NPs and liquid Ga, and the corresponding energy dispersive X-ray maps showing distribution of elements: (**a**) SEM image; (**b**–**f**) individual elemental mapping of Al, Cu, Ga, Si and Mg, respectively; (**g**–**i**) line scanning profile of elements Al, Cu and Ga, respectively.

Figure 7 shows SEM micrograph of the joint sintering bonded at 450 °C using Cu NPs and liquid Ga, and the corresponding energy dispersive X-ray maps showing distribution of elements. As shown in Figure 7a, nearly all of the nanoparticles merged into one during the bonding process, which can be called a long-range sintering process. Meanwhile, some micro-voids were left in the Cu layer after sintering bonding. The size and arrangement of sintering voids affect the mechanical strength of the joint. Moreover, Figure 7a also shows that a new phase appears along the interface between the Cu layer and substrate of SiC_p_/6063 Al MMCs. The appearance of this action layer mainly results from the high activity of Cu NPs causing the reaction of Cu and Al at the interface. The Al-Cu binary phase diagram suggests that the most possible phase at that condition is CuAl_2_ and that will be later confirmed by XRD. Figure 7b–f shows the individual elemental mapping of Al, Cu, Ga, Si and Mg in joint. As can be seen, some Al element diffused into the joint area and a little Cu element diffused into composites as well. The distribution of Mg and Si, which came from SiC_p_/6063 Al MMCs, was quite uniform in joint due to the large diffusion coefficient. Moreover, the distribution of Ga in joint was very uniform without aggregation since the diffusion velocity of Ga in Al and Cu NPs was quite high. When Cu NPs reacted with Al, they also reacted with liquid Ga to form solid solution and intermetallic compound. The line scanning profile of elements Al, Cu and Ga in Figure 7g–i also indicated that the mutual diffusion between filler metal and SiCp/6063 Al MMCs proceeded well at bonding condition, and that would be beneficial to the formation of strengthening joint.

**Figure 8:**
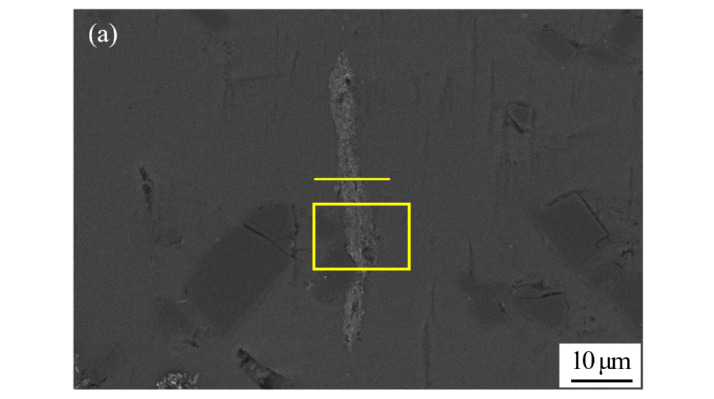

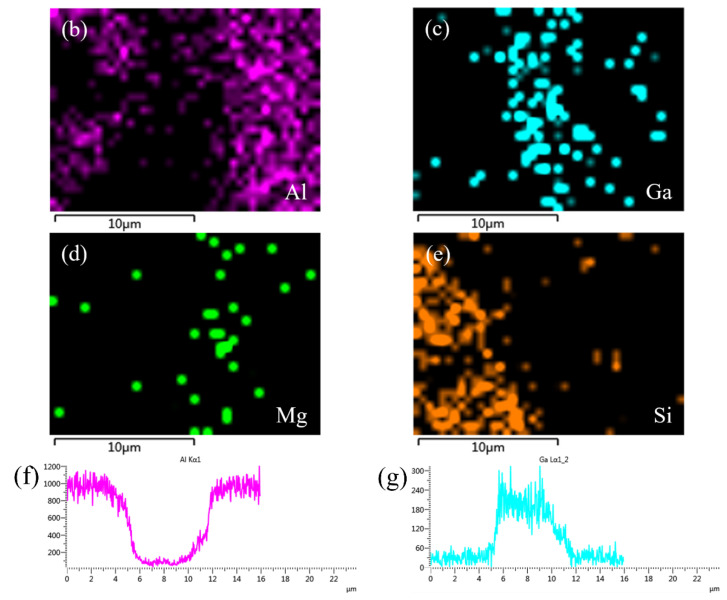
SEM micrograph at the interface of SiC_p_/6063 Al MMCs joint bonded at 450 °C using liquid Ga, and the corresponding energy dispersive X-ray maps showing distribution of elements: (**a**) SEM image; (**b**–**e**) individual elemental mapping of Al, Ga, Mg and Si, respectively; (**f**–**g**) line scanning profile of elements Al and Ga.

Figure 8 shows the SEM micrograph of the joint bonded at 450 °C using only liquid Ga, and the corresponding energy dispersive X-ray maps showing distribution of elements. Different from Figure 7d, element Ga shows an inhomogeneous distribution, as shown in Figure 8c. A large amount of Ga aggregated in the center of joint. This is because the bonding conditions, including temperature and soaking time, were not sufficient for the diffusion of liquid Ga into SiC_p_/6063 Al MMCs. Therefore, it is necessary to add some high activity material in joint to consume the residual Ga. In this research, Cu NPs were selected as the high activity material since they easily reacted with both Ga and Al in a relatively low temperature. In addition, Cu NPs have better oxidation resistance compared to other commercial metal nano particles. The distribution of Mg and Si was fairly uniform in the joint, which was similar to Figure 7. The line scanning profiles of Al and Ga also indicated that residual Ga was left in joint, as shown in Figure 8f,g. In the joint area, the residual Ga, as stated earlier, is unfavorable to the strength of the joint. Moreover, the residual Ga in joint may cause the hydrolysis of Al in humid environment, leading to the potential damage of the joint.

**Figure 9:**
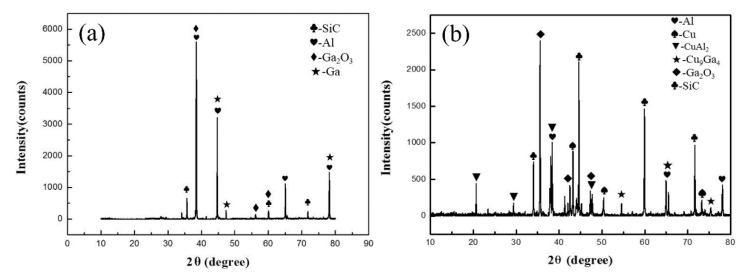
XRD results of the joint fracture bonded using: (**a**) liquid Ga at 440 °C; (**b**) Cu NPs and liquid Ga at 450 °C.

The XRD pattern of the joint fracture bonded at different conditions is presented in Figure 9. As shown in Figure 9a, when SiC_p_/6063 Al MMCs joint was bonded at 440 °C with only liquid Ga, the results showed that the main phases are Ga and Ga_2_O_3_ except the basic Al and SiC included in matrix, which further verified the above analysis results. With the addition of liquid Ga and Cu NPs in joint, the main phases in joint fracture consisted of Cu, CuAl_2_, Cu_9_Ga_4_ and Ga_2_O_3_ except the basic Al and SiC, as shown in Figure 9b. Compared with the results in Figure 9a, simple substances of Ga cannot be found in Figure 9b. The reason for that was the reaction between Cu NPs and liquid Ga in sintering bonding condition, which can consume the redundant Ga and formed the intermetallic compound Cu_9_Ga_4_ in joint. Based on the high activity of Cu NPs, the intermetallic compound CuAl_2_ was found in Figure 9b, which indicated that the interdiffusion between filler metal and base SiC_p_/6063 Al MMCs proceeded well. That intermetallic compound CuAl_2_ was also observed in Figure 7a, presenting a characteristic of dark grey phase along the interface. In both bonding conditions, Ga_2_O_3_ was detected while the aluminum oxide was not detected in joint fractures. That indicated that the Ga in joint can protect the aluminum in SiC_p_/6063 Al MMCs from the oxidation in atmosphere environment. In addition, Cu oxide was not detected in Figure 9b, which means that Ga can also protect Cu NPs from oxidation as well. Therefore, the bonding of SiC_p_/6063 Al MMCs can be realized using liquid Ga or Cu NPs and liquid Ga in air environment.

### 3.3. Mechanical Properties Analysis of the Joint

**Figure 10:**
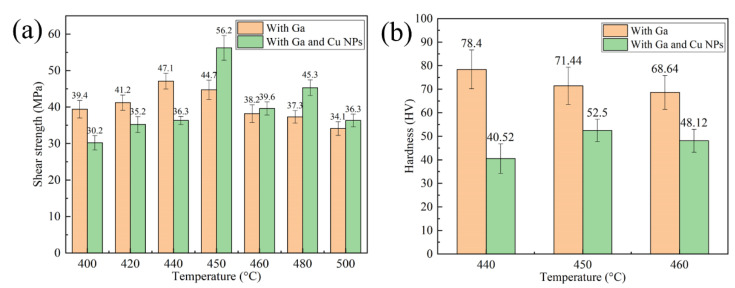
Mechanical properties of the joint bonded at different temperatures: (**a**) Shear strength of joints; (**b**) Hardness of the joints.

Figure 10 shows the mechanical properties of bonded SiC_p_/6063 Al MMCs joints by using liquid Ga, Cu NPs and liquid Ga at different temperatures under a certain pressure of 3 MPa for 2 h. As shown in Figure 10a, shear strength level of the joint bonded using Ga and Cu NPs as filler metal initially increased and then decreased. Due to the sintering inadequacy of Cu NPs at low temperature, a weak shear strength level of 30.2 MPa was presented at 400 °C. As the bonding temperature increases, the coalescence of Cu NPs leads to a steady interconnection between nanoparticles. Meanwhile, the interdiffusion between Cu NPs and SiC_p_/6063 Al MMCs substrate increased significantly with the increasing of temperature. As a consequence, shear strength of the joint increased gradually. When the bonding temperature was 450 °C, the maximum shear strength value of 56.2 MPa was achieved. Although a denser sintered structure can be obtained at a higher bonding temperature, shear strength level of the joint decreased quickly once bonding temperature exceeded 450 °C. At higher bonding temperature, a large amount of brittle intermetallic compounds such as CuAl_2_ generated along the interface between filler metal and substrate and that was harmful to mechanical property of the joint. Compared to the joint bonded with only liquid Ga, the joint bonded with Cu NPs and liquid Ga as filler metal had higher shear strength when the temperature exceeded 450 °C. Below 450 °C, the joints had lower shear strength compared with that made of liquid Ga on account of sintering inadequacy of Cu NPs.

Vickers hardness test of the joint was performed on the middle of specimens which were bonded with the temperature range of 440 °C to 460 °C. In Vickers hardness test, the loaded weight and time was set to be 300 g and 5 s, respectively. Each hardness was the average value of five measured specimen. As shown in Figure 10b, the hardness of specimen bonded with only Ga decreased gradually in the temperature range of 440 °C to 460 °C. The maximum hardness was 78.4 HV when the joint was bonded at 440 °C. With the increase of bonding temperature, Ga diffused a longer distance in joint. As a consequence, the content of Ga, which can play the role of solution strengthening, was less and less in the joint center, resulting in the decrease of hardness. The minimum hardness of the joint was 68.64 HV when the joint was bonded at 460 °C. On the other hand, the changing trend of joint hardness was consistent with that of shear strength. The maximum hardness was measured to be 52.5 HV when the joint was bonded at 450 °C. Compared with the joint bonded with only Ga, the joint bonded with Ga and Cu NPs as filler metal had lower hardness in the joint center. The main reason for that was the existence of micro-voids in the Cu layer generated during the sintering bonding process.

### 3.4. Gas Tightness Test of the Joints

**Figure 11:**
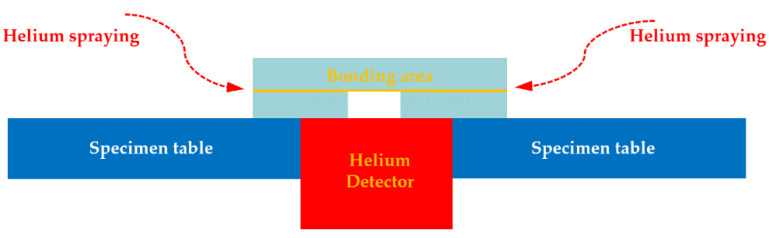
Schematic representation of gas tightness test for sintering bonded joint.

One of important application area of SiC_p_/6063 Al MMCs is electronic packaging, in which the assembly component requires strict gas tightness to protect the internal chips from potential damage caused by moist air during service. Therefore, the gas tightness test after bonding experiment was necessary to measure the leak rate of the joint, which is the only leak path of the component. In this research, helium mass spectrum detection technique was applied to measure the leak rate of the joint due to its low effective minimum detectable leak rate (5 × 10^−12^ Pa·m^3^/s) and high sensitivity. The schematic diagram of gas tightness test used in this experiment is displayed in Figure 11. Before bonding experiment, a round hole was machined on one piece of SiC_p_/6063 Al MMCs. After bonding experiment, the specimen was placed on a specimen table, in which the blind hole was located just above the detector. Meanwhile, the contact area between specimen and specimen table was sealed with sealant. After that, pure helium was sprayed on the specimen in all directions. The only path for helium entering the detector space was the bonding area.

Leak rate test of the joint was immediately performed after the bonding process. In order to test stability of the joint, the same method was carried out after one week. As a qualified component used in electronic packaging field, it is desirable that gas leakage rate keeps at 10^−10^ Pa·m^3^/s or below.

Table 1 shows gas tightness test result of the joint sintering bonded by Ga and Cu NPs at temperature range of 440 °C to 500 °C. After bonding, the specimens bonded at temperature range of 440 °C to 480 °C were qualified. Higher bonding temperature of 500 °C increased the gas leakage rate, reaching 10^−9^ Pa·m^3^/s. At higher temperature, more brittle intermetallic compounds will be generated at the interface between filler metal and SiC_p_/6063 Al MMCs. Due to different coefficient of thermal expansion between Cu layer and SiC_p_/6063 Al MMCs, the released stress will lead to damage of brittle intermetallic compounds at interface during the cooling process, causing the gas leakage. The gas tightness test after one week showed that specimens bonded at temperature range of 440 °C to 460 °C were qualified, remaining unchanged compared to the test after bonding immediately. When the specimens were bonded at 480 °C and 500 °C, the leak rates after one week were 10^−9^ Pa·m^3^/s and 10^−8^ Pa·m^3^/s, respectively. Compared with the leak rate after bonding, the leak rate of the specimens increased by an order of magnitude. The damage of the intermetallic compound layer caused by stress relief during service was the primary reason for increasing of leak rate. Though some micro-voids were observed in microstructure analysis of the joint, gas tightness of the joint sintering bonded with temperature range of 440 °C to 460 °C were qualified whether test was performed immediately after bonding or after one week. It suggests that the sintered micro-voids were disconnected in the Cu layer.

## 4. Conclusions

In this research, 15 vol.% SiC_p_/6063 Al MMCs were successfully sintering bonded using Cu nanoparticles and liquid Ga as filler metal without any flux in the air environment. The characteristics of Cu nanoparticles were analyzed at first. Based on thermophysical characteristic of Cu nanoparticles, sintering bonding technology was then applied on joining of 15 vol.% SiC_p_/6063 Al MMCs. The microstructure, shear strength, hardness and gas tightness of the joints were studied to understand the quality of the joint. The following conclusions can be drawn:

(1). For Cu NPs with the average diameter of 50 nm, two exothermic peaks can be found from DSC test. The first exothermic peak was located in the temperature range of 120 °C to 160 °C. The second broad exothermic peak was located in the temperature range of 200 °C to 320 °C. The reason for the later broad uniformity is that the Cu NPs have gone through a mild ripening process, which is a slow exothermic process. The occurrence of sintering necks in Cu NPs is the main reason for the exothermic peak. Sintering performance of Cu NPs is one of the key factors for achieving qualified joint with low gas leakage rate and high strength.
(2). When the joint was bonded at 450 °C, a large amount of pure Ga aggregated in the joint center by using only liquid Ga as filler metal. The addition of Cu NPs can consume the redundant Ga to form the intermetallic compounds in joint at the same bonding temperature. When the joint was bonded at 450 °C by using liquid Ga and Cu NPs, the main phases in joint fracture consisted of Cu, CuAl_2_, Cu_9_Ga_4_ and Ga_2_O_3_ besides the basic Al and SiC. The formation of Ga_2_O_3_ in joint can protect aluminum in SiC_p_/6063 Al MMCs and Cu NPs from the oxidation.
(3). The maximum shear strength level of 56.2 MPa can be achieved by applying bonding temperature of 450 °C. At higher temperature, although a denser sintered structure can be obtained in joint, the shear strength level decreases quickly due to the generation of large quantities of brittle intermetallic compounds such as CuAl_2_ along the interface. Compared with joint bonded with only Ga, the joint bonded with Ga and Cu NPs as filler metal had lower hardness due to the existence of micro-voids in sintering Cu layer.
(4). As the specimens sintering bonded between 480 °C and 500 °C, the released thermal stress will lead to damage of brittle intermetallic compounds along the interface during cooling or service, causing gas leakage. The specimens sintering bonded in temperature range of 440 °C to 460 °C had qualified gas tightness during the service, which can remain in the level of 10^−10^ Pa·m^3^/s. The sintering micro-voids in Cu layer had little influence on gas tightness of the joint since they were disconnected.

## Figures and Tables

**Table 1:** Gas tightness of the joint sintering bonded by Ga and Cu NPs at different temperatures.

Temperature (°C)	440	450	460	480	500
Leak rate after bonding (Pa·m^3^/s)	10^−10^	10^−10^	10^−10^	10^−10^	10^−9^
Leak rate after one week (Pa·m^3^/s)	10^−10^	10^−10^	10^−10^	10^−9^	10^−8^

## Data Availability

Not applicable.
